# Serious Games for Training Myoelectric Prostheses through Multi-Contact Devices

**DOI:** 10.3390/children9030423

**Published:** 2022-03-16

**Authors:** Rosa M. Carro, Fernando G. Costales, Alvaro Ortigosa

**Affiliations:** Department of Computer Science, Escuela Politécnica Superior, Universidad Autónoma de Madrid, Francisco Tomás y Valiente, 11. Campus de Cantoblanco, 28049 Madrid, Spain; fernandog.costales@gmail.com (F.G.C.); alvaro.ortigosa@uam.es (A.O.)

**Keywords:** special needs, training, games, adaptivity, myoelectric prostheses, rehabilitation

## Abstract

In the medical context, designing and developing myoelectric prostheses has made it possible for patients to regain mobility lost due to amputations; however, their use requires intensive training. Serious games through multi-touch devices can serve as a complement to the activities carried out during face-to-face sessions with occupational therapists and physiotherapists, as a useful resource to engage patients, especially children, and make them enjoy training. In this paper, we describe our work to support the training of myoelectric prostheses through digital serious games. Firstly, we studied the needs of children with myoelectric prostheses and the way they perform rehabilitation. Secondly, we designed specific games to support training accordingly. Thirdly, we developed a system able to generate variations of these games dynamically, adapting the elements at each round to the needs and progress of each child. The interfaces are simple, friendly, and based on tablets to favor autonomy. Finally, we assessed the potential of the use of these games for rehabilitation. Specialists in Physiotherapy, Occupational Therapy, Medicine and Special Education collaborated as experts; they agreed that SilverTouch is good for myoelectric prosthetic training and confirmed its potential to be widely used in this context.

## 1. Introduction

In recent years, scientific and technological advances are contributing to making life easier for people in general and, in particular, for those with limitations or disabilities. For example, it has been possible for individuals with physical or neurological limitations to regain movement; in addition, the use of devices as smartphones and tablets is growing exponentially. In this context, many applications exist that contribute to the development of skills for everyday life, deal with integration or socialization issues, or focus on giving support to specific training.

The goal of this work is to help those children whose upper limbs have been amputated to develop skills in the use of their myoelectric prostheses. Training in the use of this type of prostheses is usually a long and tedious task; it requires many rehabilitation sessions. Physicians, occupational therapists, and physical therapists are the ones who best know how to help children train the skills necessary to operate their myoelectric prostheses effectively; however, the hours in which each patient can receive direct attention from them are often less than desired. Yet, the more time a child can devote to training his or her prosthesis, the faster they will learn to control it. For this reason, occupational and physical therapists usually give children exercises to do at home.

In this context, it would be desirable to have support systems to complement, in an autonomous way, the work that children do with their therapists. With this goal, this work aims to provide technological support to rehabilitation, so that children who have suffered amputations can train the handling of their prostheses in an interactive, entertaining, and funny way. This objective comprises the following specific goals:

- To study the specific needs of children with amputations in their upper limbs (hands or arms) and how rehabilitation is currently performed.
- To design an application to deliver activities and games specifically designed for children to train specific skills for the use of myoelectric prostheses, with simple and friendly interfaces designed for multi-contact devices, so that children can use it independently.
- To support dynamic game generation, so that the elements of each game to be played are adapted to each user’s needs, skills, and evolution.
- To evaluate the potential use of this application in rehabilitation environments.

To carry out this work, it is necessary to have high knowledge about the use and training of prostheses, as well as the specific needs and skills to be acquired by people with this physical limitation. Nine professionals of medicine and rehabilitation have collaborated with us in this work, from the first stage, as experts: one professor of Physiology from the Faculty of Medicine at the University of Oviedo; one physiotherapist specializing in Child Therapy and his team of physiotherapists at a well-known hospital in Madrid region; and two elementary school teachers.

All professionals were involved throughout the whole process, which has followed a user-centered approach; they showed us the types of prostheses available in the market and the more appropriate exercises to train in the use of each one and also helped us to search for similar products. Complex physical devices exist for the rehabilitation of amputated limbs; however, in their experience, there are no simple applications available for patients to be specifically used for this purpose at home, autonomously, especially for children.

The experts also provided valuable input for the design of specific games and validated the prototypes created during the process, which were refined until the final version described in this article was achieved. Finally, they collaborated in testing the application and contributed to its evaluation, along with four more experts that joined this work at the evaluation stage.

The rest of the paper is organized as follows: Section 2 presents the basis of myoelectric prostheses and rehabilitation; Section 3 describes the state-of-the-art regarding the use of games for training, especially in the context of health and prostheses-related skill acquisition; Section 4 presents SilverTouch, the application built to deliver games adapted to each user and describes the three adaptive games developed so far; Section 5 shows its evaluation; finally, the discussion is presented in Section 6.

## 2. Myoelectric Prostheses

People can suffer amputations from birth, or these may have been caused later. With respect to childhood amputations, congenital ones represent 18% of the total. Traumatic amputations are caused by accidents or blows and account for 74% of the total. Finally, amputations caused by diseases or sepsis are caused by infection and represent the remaining 8%. Most of them occur after birth, which implies a change in the way of life and a need for the person to adapt to the new situation.

Prostheses are used to replace the functional deficiencies suffered by persons because of amputations. A prosthesis is an artificial limb used to replace the one amputated, and there are different types of prostheses; depending on their use, they are classified as passive or functional. Passive prostheses are those that do not replicate the functions of the real limb but are used only aesthetically. Functional prostheses reproduce the basic functions of the limb and, therefore, can move.

Depending on the type of energy they use, prostheses are classified into (i) corporeal energy prosthesis (traction, kinematics, or mechanics) and (ii) extracorporeal, electrical (myoelectric and electronic) prostheses. Electronic prostheses are used for congenital malformations; in this case, brain control over the amputated limb never existed. Myoelectric prostheses are more indicated for traumatic amputations, since the brain controls and regulates the amputated limb and can get to control the prosthesis [1]. The electrical potential generated by a muscle with contraction allows its functioning. This potential is obtained by means of electrodes placed inside the socket of the prosthesis, which is in contact with the surface of the muscle.

This work focuses on training the use of myoelectric prostheses that replace amputated limbs below the wrist (wrist disarticulation) or between the wrist and the elbow. Some myoelectric prostheses as Michelangelo [2] have multiple control functions that enable users to perform everyday activities, such as opening a tube of toothpaste, grabbing a key, inserting a credit card at a cashier, or picking up an iron for clothes. The thumb also opens, forming a natural palm shape, which can carry a plate or container. The advantages of myoelectric prostheses compared to other prostheses are:

- They allow direct communication between the central nervous stimulus and muscle contraction, provoking a response.
- The pressing force is independent of the patient’s strength or ability to move.
- They have a great functional capacity.
- The control mimics natural body functions.
- They use less body energy.
- Their power source is rechargeable lithium batteries.

The general drop-out rate of prostheses in children is very low compared to adults. Before the children can start training this type of prosthesis, a multidisciplinary team of professionals must teach them a set of guidelines for its use and care. The occupational therapists teach them to use their prosthesis properly so that they can perform all activities of daily living in the most functional possible way. A correct indication and an intense training program significantly influence the acceptance rate [3]. The objectives of training are:

- Learning to care for the prosthesis properly.
- Learning how to fit and remove the prosthesis.
- Functional training: learning how to use the prosthesis. The child must stand in front of a mirror to see the movements performed, internalize them, and correct them if necessary. Initially, he will perform broad global and simple movements.
- Dexterity and skill training: Once the patient knows and controls the movements to be performed to move his prosthesis, activities are planned to improve and combine them to achieve the greatest possible dexterity.

The importance of training has also been highlighted in ref. [4], where the significance of mastering skills before proceeding to the next set of skills is also seen as vital. Skill training is the goal of the application created: once the children can move their prosthesis, they will be helped to train the ability and dexterity of their movements.

## 3. Game-Based Training

Games have been used in many disciplines apart from entertainment [5]. In the context of education, serious games have been widely used for a wide range of purposes [6], such as making children learn while enjoying [7], helping them to develop social and adaptive skills [8], or improving professional education of people with cognitive disabilities [9], among others. In the context of health, games usually address topics such as exercise, rehabilitation, quality of life and advanced life support training [10,11].

Focusing on rehabilitation and training in health contexts, previous studies have shown that rehabilitation tasks based on a fun and playful concept provide better outcomes compared to traditional physiotherapy exercises [12]. Physiological data suggest that gaming can cause neuroplastic reorganizing, leading to the long-term retention and transfer of skills [13]. Game technologies seem to present physical and psychological benefits to pediatric patients in hospital [14]. More specifically, video games enhanced motivation and adherence in an upper limb amputee rehabilitation program [15]. Game-based interventions provide a useful addition to standard training of myoelectric prosthesis and can achieve better results, as seen in these two experiences from the same authors: ref. [16], where racing and music game genres were explored and considered as good starting points for interventions, and ref. [17], where patients with upper extremity amputation improved their neuromuscular control, strength, and coordination after using the game-based application.

However, regarding the evaluation of patient improvement, only a few studies attempt to extract game features to introduce quantitative measurements, according to the review carried out by [18] in 2020; moreover, most of the approaches found do not provide dynamic adaptation, in the sense that the game elements are adapted through the rounds according to the user-specific needs and evolution.

In addition, sometimes patients’ expectations and preferences regarding game-based prosthetic rehabilitation are different from those of researchers [19]. More recently, in ref. [20], the authors claim that the literature shows a recent surge in the number of game-based prosthetic training tools that focus on engagement and muscle training without heeding the importance of skill transfer, which is essential: myoelectric skills can be acquired through prosthetic training based on serious games only if the tasks to be accomplished allow for skill transfer.

In the work presented in this article, experts and their patients have been involved in the whole process, with the aim to provide useful myoelectric prosthesis game-based training support for those children that can move their prosthesis but still need to train the ability and dexterity of their movements. The games delivered through the application have been specifically designed to allow skill transfer; in addition, they are not the same for every patient but adapted to fit each user’s needs at each time they record each patient’s improvement, and dynamically adapt specific aspects of the games accordingly. The details of this work are given next.

## 4. SilverTouch: Dynamic Game Generation

The design of SilverTouch is oriented to the needs and skills to be acquired by the final users. The approach followed implies involving experts throughout the whole process. All details about the methodological approach, the elicitation of the application and game requirements, as well as the development and adaptivity supported, are presented next.

### 4.1. Approach

As mentioned above, the problem addressed is that training the use of myoelectric prostheses is very hard for children. Our hypothesis is that multi-contact surface adaptive games can support training for them while playing. The methodology followed in this work includes:

- Meeting experts that help us to know and understand all the details about training in the use of prostheses (e.g., movements they should practice for a correct rehabilitation) and contribute to eliciting the requirements to support this process through multi-touch games.
- Getting the experts involved in the whole process (analysis, design, evaluation of prototypes), so that the solution comprises all their expertise in this context.
- Conducting a formal evaluation of the game-based application with the experts to confirm its usefulness and suitability for rehabilitation (coverage of movements, easiness to understand, and so on). This step is crucial to avoid making a product that is not fully appropriate available to children, which could cause or increase frustration.
- Once the application is positively evaluated by the experts, making it available to occupational therapists, physicians and physical therapists, so that they can use it with children with prostheses who need training.

The goal is not to replace the rehabilitation that the children do with occupational therapists or physiotherapists, but to use the application as a complement to training in the way that the therapists advise; they will decide how to use it (children playing during face-to-face rehabilitation sessions, playing at home for certain periods of time, etc.). It is intended that children will be able to interact with the application at any time to continue training in the use of their prostheses, using their own devices in any environment.

With this purpose, the authors of this work contacted two experts in Physiology and Physiotherapy focused on children (helping children to train myoelectric prosthesis at a hospital) to tell them about their proposal, to confirm that a game-based solution for multi-contact devices could be helpful, and to get their feedback and advice on creating a useful solution; they also contacted two elementary school teachers, experts in special education, to get their feedback too. More experts joined this work: firstly, the team of physiotherapists from the hospital, who were involved in the whole process; more recently, additional rehabilitation physicians and occupational therapists have evaluated the proposal too. More details about all the tasks carried out and the role of the experts in each stage are given below.

### 4.2. Requirements

As a result of the analysis done in those meetings, the requirements elicited are summarized as follows. Each patient accesses the application through his login and password; all their interactions are stored in their profile for later analysis; the information stored in the user’s profile the first time they interact with the application includes personal data (name, surname, age, gender) and information about the hand in which they have the prosthesis (left or right), which is considered to adapt to some aspects of the games, accordingly. Once the user registers, the system stores their data in the database for later query, update, or deletion, if necessary.

The application supports several games. In each of them, the children develop a specific skill as training (more details of each are given below). Children can consult the scores obtained in every game at any time by accessing their profile, and they can also start or finish playing at any time.

As for the interaction and interface design [21], it must be simple, avoid the use of complex metaphors and provide a friendly interface that allows a comfortable use of the application, with no more buttons or elements than necessary on the screen. The application must be easy to use to ensure optimal interaction with end-users.

The logic is simple: to make it easy for the end-user to understand the game mechanism. The tasks are subdivided into simpler subtasks, while interaction is facilitated in a few steps (only those necessary for the accomplishment of the corresponding tasks). The vocabulary used in the application is simple, with keywords and phrases in a positive tone. Children interact with the application through a multi-contact interface, using a simple interaction style: drag and drop, tap, smooth movements, etc. The operation will always be the same: select option, play, or return to the previous menu. The need for the child to perform mental transformations is minimized. The buttons indicate the word of the action they represent, requiring very little effort from the user. Meaningful words and visual icons, such as arrows to move forward or back, are used.

Other non-functional requirements are modularity, scalability, security, maintainability, stability, and efficiency. The application is modular and scalable, so that each game is separated, and more games can be easily incorporated in the future. To increase data security, the data of registered users are encrypted. The code is commented and organized to facilitate later maintenance, and error control methods are implemented to avoid unrecoverable bugs in the system. The consumption of resources in the application is kept to a minimum, to favor a satisfactory user experience.

### 4.3. Game Development and Adaptivity

When meeting the experts, a paper prototype was developed as the basis for eliciting the requirements presented above, as well as for designing the games to be implemented. This prototype was further refined with their suggestions, which included those from their patients at that time. Once it was clear, a software prototype was created consisting of a mock-up emulating a real connection to the application. This interactive prototype was installed on an iPad and used to validate the functionality and appearance of the application with the experts. Once it was refined, we proceeded to implement the basis of the three configurable games designed, as well as the application to generate and display the specific rounds for each of them, able to adapt the game parameters to the characteristics and needs of the specific child to play at that time.

Although three games have been developed so far, the system allows incorporating more games in a simple way. The general functioning of the application is described as follows: The user interacts with the games through the application; each game is customized based on the user’s characteristics along with his previous and current actions while playing; all these data are stored in the user model and updated over time; the information about each child’s performance in every game played is stored centrally in that user model; this makes it possible to consider, at any time, all the interactions and achievements of a child in the games previously played for adaptation purposes in the next games to be played.

A touchscreen glove and a tablet pen are required to interact with the application. On the one hand, the glove is necessary because tablets have capacitive screens that work by detecting electrical variations on their surface. Since humans are electrically conductive, they can interact with tablets by direct skin contact, and since prostheses do not have this conductive property, it is necessary for children to wear touchscreen gloves. These gloves are knitted with conductive materials that transmit electricity from one end of the glove to the other; they are usually silver-plated nylon threads (hence the name of the application: SilverTouch). The only requirement is that some part of the skin (wrist, forearm, or elbow) meets the glove. On the other hand, the tablet pen is needed for one of the games, as an element for rehabilitative therapy. Details on its use in such therapy will be given in the description of this game. It should be noted that both objects are for sale in any online or physical electronics store, at an affordable price, allowing one of the main goals of the project to be fulfilled: supporting training at home.

The three games developed are intended to train three different functions of myoelectric prostheses, known as “Balloons”, “Dress the character” and “Connect the dots”. Each of them is described below, including its gameplay characteristics (objective and rules) as well as its rehabilitative characteristics (the function it trains).

#### 4.3.1. Game: Balloons

The playful goal of this game is to pop the highest number of balloons that appear on the screen as soon as possible. The screen shows, initially, a stage with a blue sky all over the top and a green strip of grass at the bottom. The player must rest his wrist on that strip of grass. Once he presses the start button, the balloons start to be launched.

The rehabilitative objective of the game is for the child to move their fingers in all directions without moving the rest of his arm. They are not allowed to lift their wrist from the strip of grass while playing. As the prostheses only allow movement in three fingers (thumb, index and middle), the origin of the balloons launched by the application varies according to the hand in which the child has the prosthesis (left or right).

**Figure 1:**
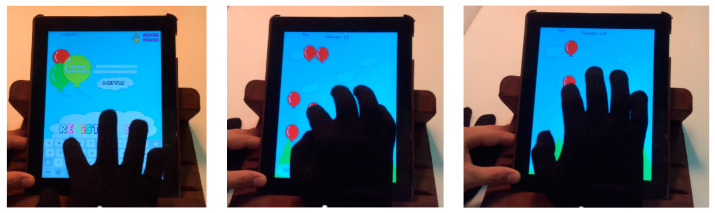
Game “Balloons”.

When the child enters the game, a button to be pressed to start appears. When they touch it, the time counter starts running, and the user can start popping balloons. The balloons move almost randomly around the screen: the lower corner on the left/right side of the screen is avoided, depending on which hand is holding the prosthesis. When the player clicks on a balloon, it explodes, making a sound that serves as feedback on the child’s action. Depending on the number of balloons popped, the level of difficulty of the game increases, maintains or decreases; this is done by increasing, maintaining, or decreasing the number of balloons presented in the screen as well as the speed at which they move. The game ends when one of the following occurs: the child has popped all the balloons or time is up; then, the score record is displayed. Figure 1 shows snapshots of a user playing “Balloons”.

To provide a greater sense of realism, the balloons are defined with some properties: friction, gravity, elasticity, and resistance. Once defined, they must be given a “stroke” to start moving, indicating the angle of the direction of the initial movement. In addition to the angle, force is assigned to define the exit velocity of the balloon. The friction property ensures that, when bouncing against an object in the environment, the balloons do not lose force. By canceling gravity, they can move around the screen in any direction. With elasticity and making use of the oval shape with which the balloons are designed (such as a fingertip) the balloons collide with each other and bounce with an angle and output force corresponding to the value returned by the functions of elasticity and angle of the object. Finally, by canceling the resistance, the balloons never slow down.

#### 4.3.2. Game: Dress the Character

This game emulates the classic game of dressing models with different clothes. The child must dress a character (chosen from those available) according to their preferences.

**Figure 2:**
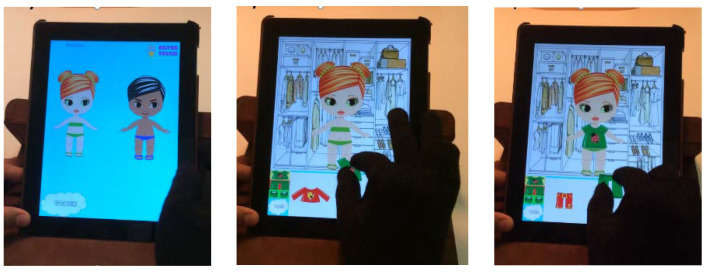
Dress the character.

As can be seen in Figure 2, once the user selects the character, the screen is split into two; the upper part shows, on a closet background, the image of the character to be dressed, while the lower part shows some tabs that group the pieces of clothing and accessories available to dress the character according to their type.

The rehabilitative objective, in this case, is for the child to be able to make the clamp (gripper) movement, that is, to grasp objects with the thumb and forefinger. To dress the character, the child needs to select the clothing using a pincer movement, as if picking it up. This movement will enlarge the garment. The child must then drag the garment to place it on the character. When the child places a correctly sized garment in the correct place, a sound is emitted as feedback on his action. In this case, there is no time limit, and no scores are assigned, according to the expert suggestions. The aim is for the children to have fun dressing the characters so that this serves as training to improve their skills in the clamp movement meanwhile; nevertheless, the size of the pieces of clothing shown to each user (i.e., the difference between their size and the character’s size) is adapted based on the information stored in his model regarding his ability with the clamp movement (needed to resize pieces). This ability may vary over time, as the user becomes more skilled; when this occurs, the information regarding this is adapted in the user model, accordingly.

#### 4.3.3. Game: Connect the Dots

**Figure 3:**
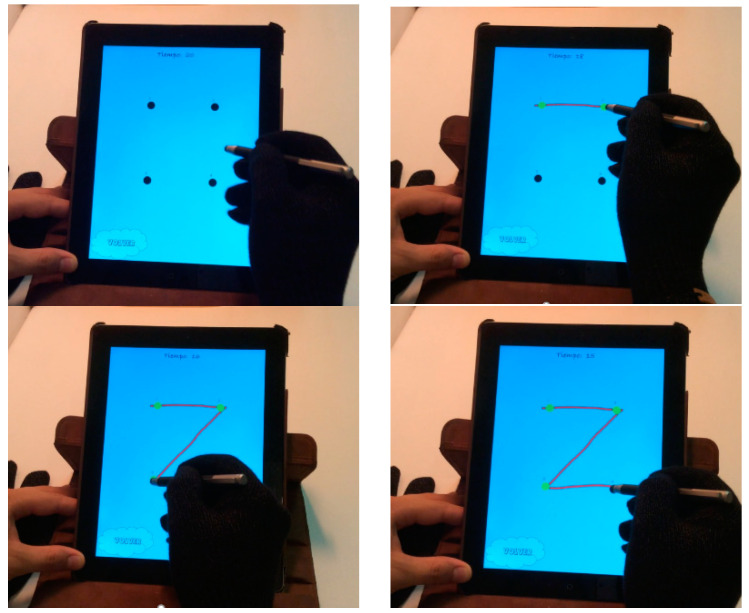
Connect the dots.

In this game, the playful objective is to connect the dots and form the shapes before time runs out. The player must use a tablet pen to connect the dots on the screen (see Figure 3).

The rehabilitation objective here is for the child to take a slightly heavy object (the pen) between his fingers, and to handle it in the directions he needs at any given moment without dropping it.

When launching the game, a message appears indicating that it is about to start; it is a timed game. When the message is accepted, the screen displays a view containing some numbered dots to be observed and connected by the child, and they must use the pen to start at dot 1, touching that dot first and connecting each dot with the next one (that is, dragging the pen from one point to the next one) until all the dots are connected sequentially. The figure is formed progressively, as the child passes through the dots: when the child joins two dots in the correct order, the line connecting them appears. The game ends when one of two things happens: the shape is completed correctly, or the time is up.

If the child forms the shape before the time is up, the level of difficulty of the game is slightly increased and the child must connect the dots of a different, more complex figure, with more dots, more distant from each other or requiring more complex movements to go from one dot to the next one. The definition of the different shapes for this game, along with their specific difficulties, was done with the help of the experts. The difficulty of the shapes presented to each child at each time is adapted (increasing or decreasing, whenever suitable and possible), firstly, according to the information stored in the user model and, afterwards, according to his performance while playing the game itself.

On the main screen, the “help and options” button allows the user to access either the rules of the games or their configuration (volume, music, and so on). All the visual elements of this application have been carefully studied together with the experts to ensure that they are the right size for children’s interactions.

The whole application has been developed for Apple iPads^TM^ running iOS7 or later as the operating system. The programming language used is Objective-C. The database uses SQL so that in the future it is easier to migrate the application to other platforms, such as Android or Windows. SQLite Manager has been used for database management. The iOS SDK kit contains all the tools needed to develop, test, execute and debug iOS applications; the two main applications used in this work are XCode and iOS Simulator. For the visual elements of the games, we used SKView objects from Sprite Kit, Apple’s 2D video game development framework. This technology allowed to develop more realistic effects, since it uses a physics engine that allows objects to interact with their environment.

Exhaustive black box and white box tests were performed to verify the correct functioning of the application, with successful results; moreover, in these types of applications, it is necessary to consider its performance, as the use of different resources could slow down or even stop it. To keep this control, CPU and memory monitoring tools were used. The use of CPU and memory was always controlled, and never exceeded the normal limits for the execution of the application on a real device. The percentage of CPU utilized was 2%, while 13.9 MB of RAM was utilized.

## 5. Evaluation

The evaluation of this work has been carried out with a multidisciplinary group of 13 experts, formed by 3 rehabilitation physicians, 6 physiotherapists, 2 occupational therapists and 2 early childhood educators specializing in special education. The evaluation was carried out as follows. Firstly, they were shown the final application developed; its operation was explained to them in 5 min. Then, they were invited to interact with it as long as they want for one week; they were encouraged to use it with their patients at that time, if any were available. Finally, they were asked to fill in a questionnaire.

The questionnaire is divided into three parts: the first one deals with the interaction and interface; the second relates to its usefulness for training different movements of myoelectric prostheses; the third one asks them about usefulness, drawbacks, and improvements, if any. The survey consists of questions with three different types of answers: a five-value Likert scale; dichotomous (yes or no as possible answers); and free-response. The results obtained are described next.

With respect to the interaction and interface design, they were asked if they consider that the application presents a friendly design; if the transition between screens seems adequate for children; if the relationship between the buttons and the actions they represent are evident; if the instructions are clear and precise; and if the games’ objectives are clearly identified. Table 1 shows the results. As can be seen, 84.62% of the experts totally agree, and 15.38% agree on its friendly design. For the next three questions, 61.54% strongly agree and 38.46% agree. None of the questions obtained neutral or disagreement answers.

In the second part of the questionnaire, the physicians, occupational therapists and physiotherapists were asked what type of movements they think can be trained using this application. Although this work is focused on training four of them (fine motor skills, proprioception, digit thumb clamp and digital flexion movement), some more were offered as possible answers. As shown in Table 2, most of the experts (88.89–100%) identified the objectives pursued by the application. In addition, the majority (66.67% and 88.89%) considered the application to be also useful for training the digital lateral slip and digital extension movements.

Finally, all the experts were asked to answer yes or no to the following questions: whether they consider the application useful for the intended purpose; whether they consider that it can be improved, and whether they find any drawback in its design or use; they had free space to explain their answers. In terms of results, 100% of them considered the application useful for its purpose. Regarding potential improvements, 53.84% do not find any, 38.46% do not know/do not answer and 7.70% (one expert) believe that one is possible; it has to do with the fact that the children would need to be able to read in order to use the application, because the functionality of some buttons is indicated in the text inside them; therefore, the proposal consists of including drawings on these buttons or adding voice instructions to make that functionality easier to understand for the youngest children. As for disadvantages, 92.30% consider that there are none, while 7.70% mention the fact that it is necessary that all the children can read, and it might be the case that the youngest ones have not learned yet. Although our application was initially oriented to children older than 6 years (age recommended for myoelectric prosthesis therapy), we keep these suggestions for future versions of the application, since, technically, there are neither limitations nor requirements to prevent its use by the youngest ones, if recommended by the experts.

## 6. Discussion

This work has contributed to myoelectric prosthesis training by providing adapted electronic game-based support to complement the work that children do with their physicians, occupational therapists and physiotherapists, so that they can train specific skills needed to use their prosthesis in an enjoyable and autonomous way.

The goals stated initially have been successfully achieved: the specific needs of children with amputations in their upper limbs and how they currently perform rehabilitation have been studied; we have designed and implemented configurable games suitable for children to train and use myoelectric prosthesis, focusing on specific skills to be acquired, according to the experts’ advice; we have designed and developed SilverTouch, an application able to dynamically generate adapted rounds of those games according to each user’s needs, skills and evolution along the time, supporting the interactions with them through multi-contact tablets; and we have evaluated the potential use of all the work done in rehabilitation environments with successful results.

As stated above, the use of serious games in the context of physical rehabilitation has already been exploited. In ref. [22] the authors describe this from both technological and clinical perspectives and discuss its advantages and drawbacks in this context. It is worth highlighting the relevance of occupational training during the fitting of myoelectric prostheses [23]. Prosthetic use training by an occupational therapist is related to the successful use of the prosthesis [3]. In ref. [24], the authors show the complexities of adopting new technologies in clinical practice; the therapists were willing to help shape the development of an upper limb training system to meet the needs of patients more readily. The conclusion of this work is that the likelihood that clinicians will adopt innovative technologies may increase by considering the needs of therapists and patients, the main actors involved in the rehabilitation process.

In this work, the results obtained suggest that SilverTouch and the adapted games it delivers can indeed help children in the training of their myoelectric prostheses, while minor modifications should be made to allow its use by children who cannot read yet. According to the evaluation made by the experts, the software developed fulfills the functional needs as expected, supporting personalized training not only of the targeted skills but also of additional ones needed to manage myoelectric prosthesis. The games delivered have been specifically designed to allow skill transfer, whose importance has been recently highlighted (versus only focusing on engagement or muscle training) [20].

In contrast to many existing approaches, SilverTouch provides dynamic adaptation: the game elements are adapted through the rounds to fit the user-specific needs at each time and his evolution; moreover, the solution developed is easily scalable. The code has been structured in separate modules, which facilitates its maintainability and extensibility. All the software has been programmed to avoid fatal errors and has been highly commented to facilitate their future maintenance by other programmers different from the authors; security and efficiency issues have also been successfully addressed.

In terms of future work, SilverTouch could be adapted to run in Android and Windows to cover more potential users. We would like to explore the incorporation of new games and additional functionality in existing ones (e.g., adding a new gesture useful to control the objects on the screen, if useful to train additional abilities). We plan to evaluate SilverTouch and its game adaptation with children at the training stage of their prosthesis, which has been postponed for two reasons: firstly, because the moment of SilverTouch final evaluation did not coincide with the rehabilitation phase in which the children in charge of the experts who participated in this work (at the hospital) were previously; and, secondly, due to COVID-19 related restrictions that affected access to hospitals later on.

We do look forward to collecting and analyzing data from patients in training stages, whenever they are available, to corroborate the positive evaluations of experts. SilverTouch should be subjected to more thorough tests and examinations to prove its effectiveness for prosthesis control improvement. Although its goal is not to replace the occupational therapists but serving as a complement for training, solid conclusions about control improvement can only be drawn after its extensive use. Given the feedback obtained from the experts, we expect the results from patients’ evaluation to follow a similar trend.

## Figures and Tables

**Table 1:** Interaction and interface questionnaire: results.

	Friendly Design	Adequate Transitions	Button-Action Coherence	Clear and Precise Instructions	Goal Identification
Totally agree	84.62%	84.62%	61.54%	61.54%	61.54%
Agree	15.38%	15.38%	38.46%	38.46%	38.46%
Neutral	0.00%	0.00%	0.00%	0.00%	0.00%
Disagree	0.00%	0.00%	0.00%	0.00%	0.00%
Totally disagree	0.00%	0.00%	0.00%	0.00%	0.00%

**Table 2:** Type of movements to be trained with the application.

Type	% Positive Answers
fine motor skills	88.89%
digital lateral sliding	66.67%
sensitivity	0.00%
proprioception	88.89%
gross motor skills	22.22%
digit thumb clamp	88.89%
thumb gripper	22.22%
digital flexion movements	100.00%
digital extension movements	88.89%

## Data Availability

Not applicable.
